# A Study on the Effect of Energy on the Development of Silkworm Embryos Using an Estrogen-Related Receptor

**DOI:** 10.3390/ijms241914485

**Published:** 2023-09-23

**Authors:** Guanwang Shen, Die Liu, Haoran Xu, Jinxin Wu, Luyu Hou, Chunyan Yang, Qingyou Xia, Ping Lin

**Affiliations:** Integrative Science Center of Germplasm Creation in Western China (Chongqing) Science City, Biological Science Research Center, Southwest University, Chongqing 400716, China; gwshen@swu.edu.cn (G.S.); liudie@email.swu.edu.cn (D.L.);

**Keywords:** estrogen-related receptor, energy, embryonic development, transgenic silkworm

## Abstract

Energy metabolism is a fundamental process in all organisms. During silkworm (*Bombyx mori*) embryonic development, there is a high demand for energy due to continuous cell proliferation and differentiation. Estrogen-related receptors (ERRs) are transcriptional regulatory factors that play crucial roles in mammalian energy storage and expenditure. Although most insects have one ERR gene, it also participates in the regulation of energy metabolism, including carbohydrate metabolism in *Drosophila*, *Aphid*, and *Silkworm*. However, no study has reported the direct impact of energy metabolism on embryonic development in silkworms. In this study, we used transgenic technology to increase silkworm (*B. mori*; Bm) BmERR expression during embryonic development and explored the impact of energy on embryonic development. We found no significant change in the quality of silkworm eggs compared to that of wild-type silkworms. However, there was an increase in the consumption of vitellin, a major nutrient in embryos. This resulted in a decrease in glucose content and a significant increase in ATP content. These findings provide evidence that the acceleration of energy metabolism promotes embryonic development and enhances the motility of hatched silkworms. In addition, these results provide a novel perspective on the relationship between energy metabolism and embryonic development in other insects.

## 1. Introduction

The transcriptional control of cellular energy metabolism pathways is achieved through the coordinated action of multiple transcription factors and associated co-regulatory factors. Nuclear receptors are members of the transcription factor superfamily, which mediate the signaling of hormones, nutrients, metabolites, and redox signals into specific metabolic gene programs, and thus play important roles as regulatory factors in cellular energy generation. They can convert signals from hormones, nutrients, and metabolites into specific gene expression networks to meet the energy demands for different physiological cues [1].

Most nuclear receptors are ligand-dependent, but some are referred to as orphan receptors because no natural ligands have been discovered for them. Estrogen-related receptors are classified as orphan receptors. In vertebrates, there are three subtypes of estrogen-related receptors (ERRs), namely ERRα, ERRβ, and ERRγ [2]. ERRs are widely distributed across vertebrates and play crucial roles in regulating various processes related to nutrient breakdown and energy metabolism in mammals [1,3,4,5,6]. Their expression is highly correlated with mitochondrial biogenesis [7] and oxidative phosphorylation, participating in lipid metabolism, glucose metabolism, and oxidative phosphorylation pathways [8]. In glucose metabolism, ERRs mainly regulate glycolysis, gluconeogenesis pathways, and enzymes related to the tricarboxylic acid cycle in mitochondria, thereby regulating organismal glucose metabolism [9]. Abnormal ERR overexpression has been observed in cancer cells and has a significant impact on glycolytic gene expression and glucose uptake [10]. In lipid metabolism, enzymes involved in fatty acid beta-oxidation pathways, such as acetyl-CoA dehydrogenase and propionyl-CoA carboxylase, are target genes of ERRs [11].

Research advancements have established that ERR genes are also present in insects [12,13], where they play crucial roles [14,15,16,17,18]. In aphids, the estrogen-related receptor (ERR) can bind to the ERR response elements in the promoters of phosphofructokinase and pyruvate kinase, thereby modulating glycolysis. The overexpression of ERRs leads to a substantial increase in the gene expression of these two enzymes, which serve as rate-limiting steps in the glycolytic pathway. Additionally, a decrease in the expression level of ERRs in viviparous female aphids causes a reduction in the expression of glycolysis-related genes, ultimately resulting in a decline in offspring production [19]. They are involved in the regulation of almost all processes related to carbohydrate metabolism in *Drosophila* [20,21,22,23]. Mutations in *Drosophila* ERRs (DroERRs) lead to the severe impairment of energy metabolism, resulting in elevated trehalose levels in the larvae, significantly reduced ATP levels, and the downregulated expression of the genes involved in carbohydrate metabolism [22,23]. In addition, the demonstration of DroERR involvement in both HIF (hypoxia-inducible factor)-dependent and HIF-independent pathways as a steroid hormone receptor indicates the essential role of ERRs in hypoxia [24]. Moreover, ERRs are also involved in spermatogenesis in *Drosophila*. The knockdown of ERRs in the testes disrupt testis development and dysregulate the genes involved in sperm production, resulting in a significant reduction in sperm count and a decline in male *Drosophila* [25]. Our previous studies have shown that the ERR in silkworms (BmERR) can bind to estrogen response elements on the trehalase gene promoter, thereby upregulating the expression of trehalase in the midgut. This suggests that trehalose accumulation in *Drosophila* larvae may be related to the impaired trehalose breakdown caused by an ERR deficiency [26].

Silkworms (*Bombyx mori*) are holometabolous insects that undergo metamorphosis and reproduce by laying eggs [27,28]. Embryonic development in silkworms involves an intensive catabolic metabolism whereby the nutrients stored within the eggs are extensively utilized to provide energy for the complete development of the embryo [29]. However, no study has reported the direct impact of energy on embryonic development in silkworms. Research on the development of silkworm embryos has been ongoing for many years, and the entire morphology process of silkworm embryo development is relatively clear. Silkworm embryo development requires a significant amount of energy for proper support. However, there is currently no evidence regarding the direct effect of energy metabolism on silkworm embryo development. The regulation of cellular energy metabolism by ERRs is almost indisputable in both vertebrates and silkworms. In this study, we used transgenic technology to increase BmERR expression during silkworm embryonic development and explore the impact of energy on embryonic development. The findings of this study will provide a novel perspective on the relationship between energy metabolism and embryonic development in other insects.

## 2. Results

### 2.1. Overexpression of BmERRs in the Eggs of Transgenic Silkworms

**Figure 1:**
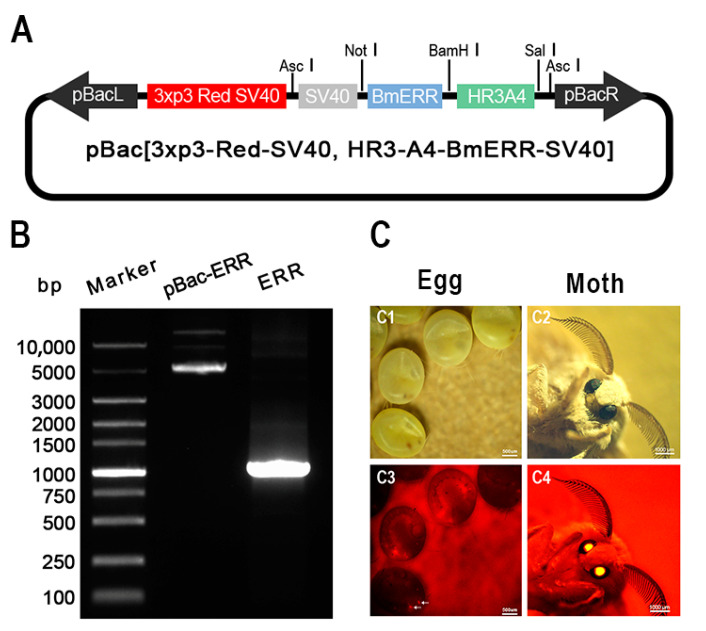
Generation of transgenic silkworms. (**A**) Schematic diagram of the transgenic vector (piggyBac [3xp3-Red-SV40, HR3-A4-BmERR-SV40] vectors) for overexpressing BmERRs. pBacL and pBacR represent the left and right arms of the transposon, respectively. 3xp3-Red-SV40 indicates the transgenic selection marker. HR3-A4 is the promoter. (**B**) Agarose gel electrophoresis analysis: Lane 1 shows the pBac-ERR, which is the transgenic plasmid piggyBac-[3xp3-Red-SV40, HR3-A4-BmERR-SV40]. Lane 2 shows the PCR-amplified ERR band obtained from piggyBac-[3xp3-Red-SV40, HR3-A4-BmERR-SV40]. The length of the ERR band is approximately 1300 bp. (**C**) The isolation of generation G1 transgenic silkworms through the detection of DsRed expression in the compound eyes of eggs (as shown by the white arrow in Figure 1C C3) and moths. (**C1**–**C4**): Positive transgenic silkworm larvae screened under a fluorescence microscope; (**C1/2**) white light images of the transgenic *B. mori* eggs/moths; (**C3/4**) fluorescence images of the transgenic *B. mori* eggs/moths.

To investigate the effect of energy metabolism on the development of *B. mori* embryos, we designed and constructed a transgenic overexpression plasmid (pBac-Hr3-A4-BmERR-SV40) using the PiggyBac transposon system. A4 is a cytoplasmic actin gene present in all eukaryotic cells. Silkworm A4 (BmA4) may be functionally equivalent in other animals [30]. Hr3 (homologous region 3) is the nuclear polyhedrosis virus of the silkworm, which has been proven to greatly enhance the transcriptional activity of the A4 gene promoter in the silkworm [31,32]. Using the HR3-A4 promoter to drive gene expression in silkworms is a long-term research goal of our research team [33,34]. The plasmid used the Hr3-A4 promoter to drive the expression of the BmERR gene. In addition, a 3xp3 eye-specific promoter was used to drive the expression of a red fluorescent protein, which served as a positive transgenic selection marker (Figure 1A). We tested these plasmids to confirm the presence of BmERRs, the amplification of BmERRs using a PCR, and that the PCR yielded bands of the expected sizes (Figure 1B). After injecting the transgenic vectors into the silkworm eggs, red fluorescence was detected in the compound eyes of the G1 eggs and moths (Figure 1C), which was named OE-ERR.

**Figure 2:**
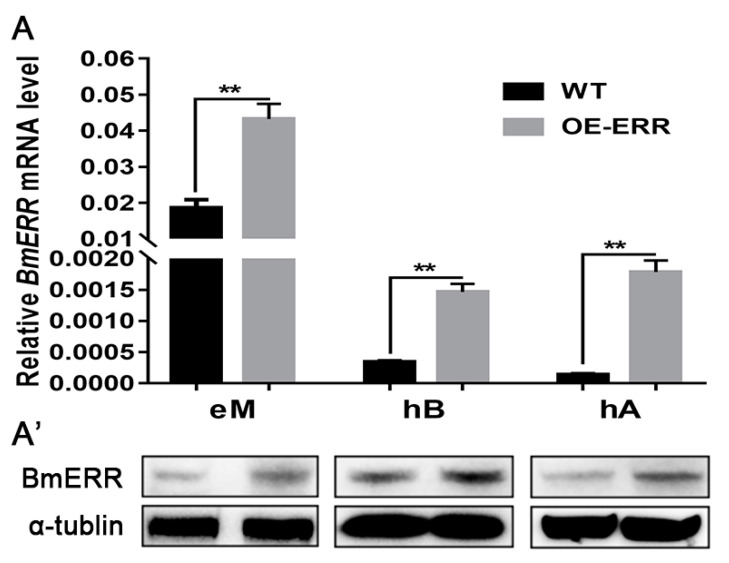
BmERR expression in transgenic silkworm embryos. (**A**) Detection of BmERR gene expression. (**A’**) Detection of BmERR protein expression; eM: eggs of moth; hB: before hatching of silkworm embryos; hA: after hatching of silkworm embryos; WT: wild-type *B. mori*; OE-ERR: overexpression of BmERRs in transgenic *B. mori*; **, *p* < 0.01; *t*-test.

To analyze the incremental expression of BmERRs during the embryonic stage of transgenic *B. mori*, RNA was extracted from the eggs, pre-hatching embryos, and post-hatching embryos of various embryo developmental stages. qRT-PCR and Western blotting techniques were used to assess the expression levels of BmERRs. The results indicated a significant increase in the levels of BmERRs in the embryos of transgenic silkworms compared to the wild type at all three time points, both at the gene (Figure 2A) and protein levels (Figure 2A’). These findings confirmed the successful incremental expression of BmERRs in silkworm embryos.

### 2.2. Impact of Incremental BmERR Expression on Silkworm Eggs

**Figure 3:**
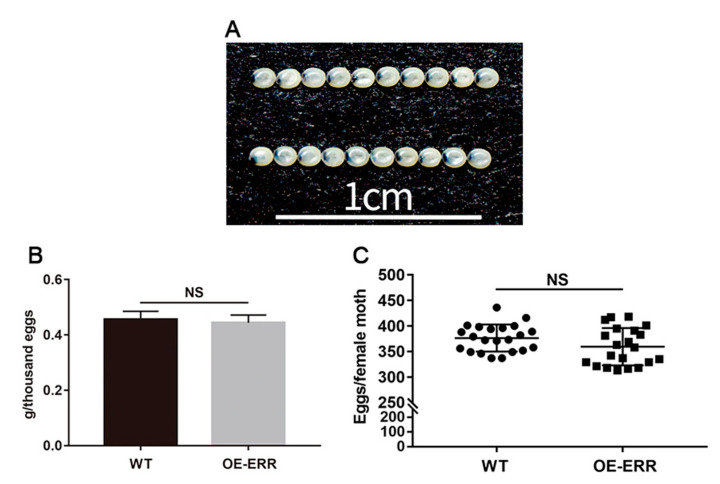
Effects of the incremental expression of silkworm estrogen-related receptors (BmERRs) on silkworm embryo quality. (**A**–**C**) The size (**A**), weight (**B**), and number (**C**) of eggs laid by female moths; WT: wild-type *B. mori*; OE-ERR: overexpression of BmERRs in transgenic *B. mori.* NS: not significant (*t*-test).

To assess the influence of incremental BmERR expression on embryonic development, we conducted a comparative analysis of the size, weight, and quantity of transgenic and wild-type silkworm eggs. The results demonstrated no significant differences in the size (Figure 3A), weight (Figure 3B), or number of eggs laid by female moths (Figure 3C), indicating that increased BmERR expression does not affect silkworm embryo quality.

### 2.3. The Impact of the Increased Expression of B. mori Estrogen-Related Receptors (BmERRs) on Embryonic Energy Metabolism

**Figure 4:**
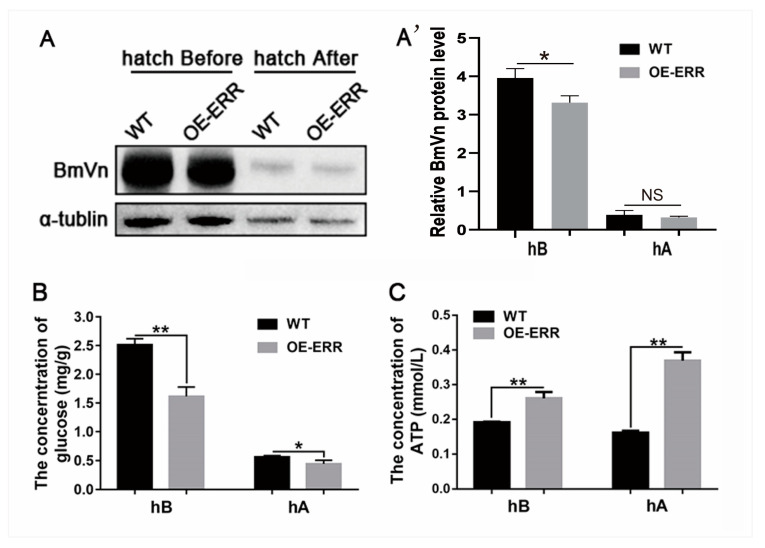
Impact of incremental expression of silkworm estrogen-related receptors (BmERRs) on energy metabolism. (**A**) Detection of BmERR protein expression in oviposited eggs using Western blotting. (**A’**) Quantitative analysis of BmVn protein level using Western blotting. (**B**) Glucose content in embryos before and after hatching. (**C**) ATP content in embryos before and after hatching. eM: eggs of moth; hB: before hatching of silkworm embryos; hA: after hatching of silkworm embryos; WT: wild-type *B. mori*; OE-ERR: overexpression of BmERRs in transgenic *B. mori*. *, *p* < 0.05, **, *p* < 0.01; *t*-test, NS: not significant (*t*-test).

Since embryonic development in *B. mori* primarily relies on the consumption of vitellin stored in eggs to provide energy [35,36], we first examined the level of BmVn (vitellin) in transgenic silkworms before and after hatching. We found that transgenic embryos with increased BmERR expression showed a decrease in BmVn content before hatching, whereas the remaining BmVn content in the embryos after hatching was approximately the same (Figure 4A,A’), indicating that increased BmERR expression accelerated the consumption of BmVn by the embryos. Furthermore, we examined the levels of glucose and ATP in the embryos before and after hatching and found that, compared to the wild type, the transgenic embryos had significantly reduced glucose content (Figure 4B) but significantly increased ATP content (Figure 4C). This indicates that increased BmERR expression in embryos promotes the conversion of glucose into ATP by consuming BmVn, which serves as the main source of nutrients and energy during the silkworm embryonic development stage.

### 2.4. Impact on Energy Metabolism and Embryonic Development in Silkworms

**Figure 5:**
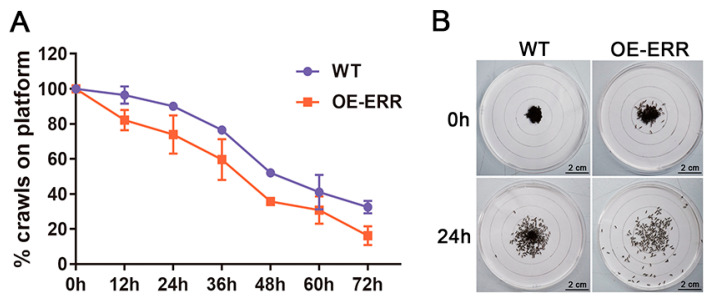
Silkworm comparative vitality test. (**A**) The percentage of silkworms that remained within the starting plane was measured at different time intervals after embryo hatching, with measurements taken every 12 h. The data are presented using colored bars: blue represents the wild-type (WT) and red represents the transgenic (OE-ERR) silkworms. (**B**) The activity trajectories of both the wild-type and OE-ERR transgenic silkworms within the plane were tracked and displayed at the 24 h mark. WT: wild-type *B. mori*; OE-ERR: overexpression of BmERRs in transgenic *B. mori*.

As previously mentioned, the incremental expression of BmERRs did not affect the size or the weight of the silkworm eggs. Consequently, we conducted a comparative test of the vitality of newly hatched silkworms and assessed their movement ranges and quantities. The results revealed that, at each time interval, the number of transgenic silkworms remaining within the starting circle was lower than that of wild-type silkworms (Figure 5A). Furthermore, at the 24 h mark, the transgenic silkworms exhibited a more dispersed crawling pattern (Figure 5B). These findings suggest that transgenic silkworms demonstrate greater vitality than their wild-type counterparts. Taken together, the silkworm embryos’ overexpression of BmERRs improves the efficiency of glucose conversion to ATP, increases the consumption of vitellogenin in the silkworm embryos, and leads to the hatching of silkworms with more active exercise ability.

## 3. Discussion

In this study, we successfully utilized the HR3-A4 promoter to overexpress BmERRs during the silkworm embryonic development stage, which accelerated the consumption of nutrients and increased ATP content with a decrease in glucose content, which ultimately led to more active behavior in the hatched larvae. The transgenic silkworm larvae moved farther on the plate than those of the wild type because of excessive nutrient consumption due to BmERR overexpression, and the transgenic larvae felt hungrier and moved farther on the plate in search of food compared to the wild-type silkworms.

Silkworm embryonic development is an intensive process requiring substantial amounts of energy and materials [37]. BmVn, a major nutrient in silkworm embryos, providing carbohydrates, fats, amino acids, and other substances for embryonic development, serves as the main source of nutrients and energy during the developmental stage [35,36]. Carbohydrates play a crucial role as primary and direct sources of nutrition for silkworm embryonic development [29,38]. In normally developing silkworm embryos, glycogen serves as an immediate energy source because it is decomposed and utilized. Some glycogen reserves originate from BmVn molecules that undergo glycosylation before entering the ovaries. Glycosylation involves the attachment of N-linked mannose oligosaccharides, which facilitates their transport into embryos [35,36]. Glycogen reserves serve as fundamental materials for embryonic energy metabolism. The accelerated consumption of BmVn observed after BmERR overexpression during the embryonic stage may be attributed to the intricate relationship between Vn and glycogen reserves. Our previous study found that the interference of BmERRs in female silkworms’ fat body induced *BmVg* transcription, resulting in an increase in egg weight relative to the control [39]. This may be due to the expression of the *A4* gene in all cells of the silkworm, resulting in the overexpression of the A4 promoter-driven BmERR in other tissues (e.g., fat body) of transgenic silkworms [32]. Although the content of BmVn in silkworm eggs has increased, the consumption of BmVn in eggs has also accelerated, ultimately resulting in no significant change in the egg quality of transgenic silkworms compared with wild-type silkworms. Further studies are needed to address this issue, such as using a silkworm-ovary-specific endogenous gene promoter to drive BmERR-specific overexpression in the ovary to study the effect of BmERRs on the development of silkworm embryos. In this study, our findings were limited to explore the effect of energy on silkworm embryonic development.

Carbohydrate metabolism is a fundamental metabolic process during silkworm development [29,39,40,41]. In the glycolytic pathway, hexokinase (HK) facilitates glucose phosphorylation to form glucose-6-phosphate. Increased HK activity plays an important role in the embryonic development of silkworms [42]. In our previous study, we found that the expression level of BmERRs gradually increases with the development of silkworm embryos, and BmERRs bind to the estrogen-related receptor response element (ERRE) on the phosphofructokinase gene (PFK, a key rate-limiting enzyme in the glycolytic pathway) promoter to regulate the expression of PFK, which regulates the glycolysis process of silkworm embryos. Interference with BmERR expression during the embryonic stage leads to a reduced expression of three key rate-limiting enzymes in the glycolytic pathway: PFK, HK, and pyruvate kinase (PK). This results in elevated glucose levels and a delay in silkworm hatching [43]. In the present study, we explored the relationship between estrogen-related receptors, energy metabolism, and silkworm embryonic development, with a specific focus on their regulatory roles in the glycolytic pathway during the embryonic stage. By overexpressing BmERRs in silkworms, we demonstrated their regulatory function in the glycolytic process, facilitating the conversion of glucose to ATP and promoting the successful transition from silkworm embryos to larvae. Previous studies have reported that the DroERR regulates the transition from pupa to adult by promoting glucose oxidation and fat synthesis: *Drosophila* mutation with a loss of ERR function in adults exhibits an accumulation of triglycerides and reduced glucose levels. Although these adults can survive, they exhibit decreased locomotor ability and increased sensitivity to hunger [20]. The insect embryo hatches and transforms into a larva, and its metabolism is carefully adjusted to promote exponential growth. The embryonic development state is metabolized in contrast to larvae, which depends on internal energy reserves. This suggests that ERRs in insects may be involved in metamorphosis by regulating metabolism.

Notably, silkworms undergo diapause during the embryonic stage, which is a period of developmental arrest. Eggs can be classified as diapausing or non-diapausing [38,44,45,46,47]. Non-diapausing eggs develop and hatch approximately ten days after laying. In contrast, diapause eggs enter a period of dormancy after approximately one week of embryonic development. During diapause, a significant amount of glycogen is converted to sorbitol and glycerol, and embryonic metabolism ceases [48,49]. When diapause terminates and development resumes [49,50], sorbitol and glycerol are converted back to glycogen and glucose, respectively, as preparatory steps for further development [28,37,49,50,51,52,53,54]. Diapause hormones play a vital role in insect diapause by regulating the conversion of trehalose into ovarian glycogen [37,51,52,55,56,57,58]. Conversely, BmERRs regulate trehalase activity, promote the conversion of trehalose to glucose, and regulate ATP production [26]. We found that the overexpression of BmERRs during silkworm embryo development increased the consumption of vitellogenin in silkworm embryos, improved the efficiency of glucose conversion to ATP, and led to the hatching of silkworms with more active exercise ability. This indicates that there may be a potential connection between silkworm embryonic diapause and BmERRs, and further studies on the relationship between diapause hormones and BmERR expression are necessary. Nonetheless, these questions remain an important area for extensive investigation. The study of the silkworm diapause mechanism has been ongoing for nearly a century, and addressing these questions will provide valuable insights into the intricate mechanism of embryonic diapause in insects.

## 4. Materials and Methods

### 4.1. Insects

For this experiment, the D9L strain of silkworms obtained and maintained at our institution was used. The silkworms were fed a diet consisting of fresh mulberry leaves and were kept at a controlled temperature of 25 ± 1 °C. A 12 h light/dark cycle was used for the feeding schedule.

### 4.2. Construction of Transgenic Silkworms

A transgenic vector, piggyBac (3×p3-DsRed-SV40 and HR3-A4-BmERR-SV40), was prepared. Primers for seamless cloning were designed to amplify the HR3-A4-BmERR-SV40 fragment from the psl 1180-HR3-A4-BmERR-SV40 cell expression vector using a PCR. The amplified fragment was integrated into a transgenic vector via homologous recombination. The PCR amplification conditions were as follows: initial denaturation at 98 °C for 3 min, followed by cycling at 98 °C for 10 s, 56 °C for 20 s, and 72 °C for 40 s, repeated for a total of 35 cycles. The final extension step was performed at 72 °C for 5 min. PCR amplification was conducted using PrimeSTAR^®^ Max DNA Polymerase (TAKARA, Kyoto, Japan), as recommended by the manufacturer, and the seamless cloning process was performed using the Seamless Cloning Kit (Abm, Shanghai, China).

The D9L insects laid eggs, which were microinjected with a mixture of transgenic recombinant plasmids (400 ng/μL) and auxiliary plasmids (400 ng/μL) within 2 h. The injected eggs were incubated at a temperature of 25 °C and provided with fresh mulberry leaves for feeding. Subsequently, the silkworm eggs and moths were screened for DsRed-positive expression using fluorescence microscopy. The transgenic silkworm line selected during the screening process was designated as OE-ERR for future experiments.

### 4.3. RNA Extraction and cDNA Synthesis (Primer Sequences and Amplification Conditions)

Embryos from different developmental stages of both the wild-type D9L and the transgenic BmERR-overexpressing silkworms were collected and immediately frozen in liquid nitrogen. The frozen samples were stored at −80 °C for later use. Clean pestles and mortars were wrapped in aluminum foil and dried at 120 °C for 3 h. After pre-cooling with liquid nitrogen, the tissues were ground thoroughly. Total RNA was extracted using the TRIzol extraction kit (Invitrogen, Carlsbad, CA, USA). DNase I digestion was performed (TAKARA) to avoid genomic contamination. The concentration and purity of the extracted RNA were determined through spectrophotometry. The cDNA was synthesized following the instructions provided by the M-MLV Reverse Transcription Kit (Promega, Madison, WI, USA). The RNA samples were stored at −80 °C, and the synthesized cDNA was stored at −20 °C for future use. A real-time quantitative PCR was performed.

A real-time quantitative PCR was conducted using SYBR Premix Ex Taq™ (TAKARA) and the ABI StepOne v2.1 Sequence Detection System (Applied Biosystems, Foster City CA, USA) to evaluate the expression level of BmERRs (GenBank: KT268294). The internal control used was the silkworm translation initiation factor 4A (BmTIF4A, NM_001043911.1), and the relative expression level of the target gene was determined using the 2^−ΔΔCt^ method. The PCR amplification program included an initial denaturation step at 95 °C for 5 min, followed by 35 cycles of denaturation at 95 °C for 3 s and extension at 60 °C for 30 s. The specific primers used for the fluorescence quantitative PCR were as follows: BmERR F 5′-CGCCGACCTGTACGACC-3′, R 5′-CACGCCCGACACCTGTAGAAA-3′, BmTIF4A F 5′-TTCGTACTGGCTCTTCTCGT-3′, R 5′-CAAAGTTGATAGCAATTCCCTA-3′.

### 4.4. Protein Extraction and Immunoblotting

The tissue materials were thoroughly ground in a pre-chilled mortar using liquid nitrogen and an appropriate amount of PBS solution was added. Protein degradation was prevented by adding the protease inhibitor PMSF to a final concentration of 1 mM. The mixture was incubated at 4 °C for 1–2 h. The centrifuge was pre-cooled to 4 °C, and the supernatant was collected after centrifugation at 12,000× *g* for 15 min. Protein concentration was determined using a BCA Protein Concentration Assay Kit (Beyotime, Shanghai, China). Protein samples were separated using sodium dodecyl sulfate-polyacrylamide gel electrophoresis on 12% (*w*/*v*) polyacrylamide gels. After electrophoresis, proteins were transferred to PVDF membranes. The membranes were blocked with 5% non-fat milk in TBST and incubated with primary antibodies specific for BmERRs, BmVn, or α-tubulin. Horseradish peroxidase (HRP)-conjugated goat anti-rabbit IgG (Beyotime, Shanghai, China) was used as the secondary antibody for BmERRs and BmVn, whereas goat anti-mouse IgG was used for α-tubulin. The antibodies were diluted in 2% non-fat milk in TBST solution at a 1:10,000 ratio. Protein immunoblotting was performed using the Clinx ChemiScope Mini 3400 imaging system (Shanghai Institute of Scientific Research, Shanghai, China).

### 4.5. Phenotypic Statistics

The sizes of the wild-type D9L and the transgenic silk moth embryos overexpressing BmERRs were measured. For comparison, ten silkworm eggs of each type were arranged in longitudinal rows. The thousand-grain weights of the wild-type and the transgenic silkworm eggs (*n* = 3) were measured and recorded. The number of silkworm eggs laid by the transgenic and wild-type female moths was collected and quantified.

### 4.6. Newly Hatched Silkworm Activity Test

In this experiment, a concentric circle plane was constructed using white paper, with the smallest circle serving as the starting point for the clustered newly hatched silkworms. Silkworms exhibit locomotive behavior when searching for food. The number of newly hatched silkworms remaining within the starting circle was recorded at 12 h intervals for a total duration of 72 h, and the percentages were calculated.

### 4.7. Detection of Glucose and ATP Levels

Fresh tissue material (100 mg) or material stored in liquid nitrogen was collected. The material was homogenized on ice according to the manufacturer’s instructions and the supernatant was obtained after centrifugation. A 96-well microplate was used to mix the corresponding reagents and samples, and absorbance was measured at 340 and 570 nm. Glucose levels were determined using a Glucose Detection Kit (LEAGENE, Beijing, China) according to the manufacturer’s instructions, whereas ATP levels were determined using an ATP Detection Kit (Sigma-Aldrich, Taufkirchen, Germany).

### 4.8. Statistical Analysis

Statistical values are reported as mean ± standard error of the mean (SEM). A Student’s *t*-test was used to compare mean values against the following significance thresholds: * *p* < 0.05, ** *p* < 0.01, and *** *p* < 0.001. Statistical analyses were conducted using GraphPad Prism 7.0 software (GraphPad Software, San Diego, CA, USA). Graphs were constructed using Adobe Photoshop CC 2019 and Adobe Illustrator CC 2017.

## 5. Conclusions

Silkworm embryonic development is an intensive process requiring substantial amounts of energy and materials. BmVn serves as the main source of nutrients and energy during this developmental stage. ERRs are widely present in vertebrates and play crucial roles in regulating various processes related to nutrient decomposition and energy metabolism in mammals. The overexpression of BmERRs in silkworm embryos improves the efficiency of glucose conversion to ATP, increases the consumption of vitellogenin in silkworm embryos, and leads to hatched silkworms with greater motility. The findings of this study provide a novel perspective on the relationship between energy metabolism and embryonic development in insects.

## Data Availability

Not applicable.
